# Associations between Variants in IL-33/ST2 Signaling Pathway Genes and Coronary Heart Disease Risk

**DOI:** 10.3390/ijms151223227

**Published:** 2014-12-15

**Authors:** Fangqin Wu, Mei’an He, Qiang Wen, Wencai Zhang, Jinhua Yang, Xiaomin Zhang, Tangchun Wu, Longxian Cheng

**Affiliations:** 1 Department of Cardiology, Union Hospital, Tongji Medical College, Huazhong University of Science and Technology, 1277 Jiefang Dadao, Wuhan 430022, China; E-Mails: wufangqinnice@gmail.com (F.W.); tjwenqiang@126.com (Q.W.); zwc.8425@163.com (W.Z.); jinhua0371@163.com (J.Y.); 2 Institute of Occupational Medicine and the Ministry of Education, Key Lab of Environment and Health, School of Public Health, Tongji Medical College, Huazhong University of Science and Technology, 13 HangKong Road, Wuhan 430030, China; E-Mails: hemeian@hust.edu.cn (M.H.); mingxz117@hust.edu.cn (X.Z); wut@mails.tjmu.edu.cn (T.W.)

**Keywords:** coronary artery disease, ST2, IL-33, IL-1RAcP, signaling pathway, gene

## Abstract

The IL-33/ST2 signaling pathway plays an important role in coronary artery disease (CHD); however, few studies have explored how variants in *IL-33/ST2* genes influence CHD risk. Here, we examined the association between genetic variants in *IL-33*, *ST2*, and *IL-1RAcP* of the *IL-33/ST2* axis and the risk of CHD. We conducted a case-controlled study with 1146 CHD cases and 1146 age- and sex-frequency-matched controls. Twenty-eight single nucleotide polymorphisms (SNPs) in *IL-33*, *ST2*, and *IL-1RAcP* were genotyped by Sequenom MassArray and TaqMan assay. Logistic regression was used to analyze these associations. The SNP rs4624606 in *IL-1RAcP* was nominally associated with CHD risk. The AA genotype was associated with a 1.85-fold increased risk of CHD (95% confidence interval (CI) = 1.01–3.36; *p* = 0.045) compared to the TT genotype. Further analysis showed that AA carriers also had a higher risk of CHD than TT + TA carriers (odds ratio (OR) = 1.85; 95% CI = 1.85–3.35; *p* = 0.043). However, no significant association was observed between variants in *IL-33/ST2* genes and CHD risk. Further studies are needed to replicate our results in other ethnic groups with larger sample size.

## 1. Introduction

Coronary heart disease (CHD) is a complex chronic inflammatory process mediated by the innate and acquired immune systems and involves pro- and anti-inflammatory cytokines [1]. Evidence indicates the interleukin 33/interleukin 1 receptor-like 1 (*IL-33/ST2*) axis participates in various fields of cardiovascular disease [2,3,4,5,6,7]. IL-33 is a member of the interleukin-1 family of cytokines, and ST2, which consists of a transmembrane ligand (ST2L), a soluble component (sST2), and ST2V, is the receptor for IL-33 [8]. IL-33 and ST2L form a heterodimeric receptor complex with IL-1R accessory protein (IL-1RAcP) [9,10]. By interacting with ST2L, IL-33 prevents cardiomyocyte apoptosis and attenuates myocardial fibrosis and myocyte hypertrophy [4]. *In vivo*, IL-33 significantly attenuates atherosclerotic cardiovascular disease in ApoE^−/−^ mice on a high-fat diet via the induction of IL-5 and oxidized low-density lipoprotein antibodies. The IL-33 decoy receptor, sST2, alters the atheroprotective activity of IL-33 [2]. In contrast, other studies have reported that IL-33 promotes adhesion molecules and inflammatory activation in human endothelial cells and may, in such way, enhance the development of atherosclerotic lesions in the vessel wall [11,12]. The mechanisms of these contradictory effects of IL-33 are not yet completely understood yet. Clinically, IL-33 serum levels were elevated in patients with heart failure and associated with coronary in-stent restenosis and mortality in patients with ST elevation myocardial infarction [13,14,15,16]. Furthermore, sST2 has been demonstrated as a strong prognostic biomarker in patients with heart failure and myocardial infarction [3,5,17,18,19].

Despite powerful biological and prognostic data describing the role of IL-33/ST2 signaling in the development of CHD, there is little information regarding the significance of *IL-33/ST2* genetic variants in CHD. Recently, Tsapaki *et al.* revealed that two polymorphisms in the distal promoter region of *ST2* were associated with susceptibility to severe CHD [20]. In addition, polymorphisms in genes involved in the IL-33/ST2 pathway have been associated with several immune and inflammatory diseases [21,22,23,24,25,26,27,28,29,30,31,32,33].

Therefore, considering the role of inflammation in CHD, we hypothesized that polymorphisms in *IL-33/ST2* might be associated with CHD risk. To test this hypothesis, we performed a genetic association analysis of *IL-33/ST2* and CHD risk in a case-control study of the Han Chinese population.

## 2. Results and Discussion

### 2.1. Characteristics of the Case and Control Subjects

The general characteristics of the case-control study population are presented in Table 1. Age and sex were similar for case and control subjects. Traditional CHD risk factors, such as body mass index (BMI) and fasting blood glucose (FBG), were significantly higher in CHD cases than in control subjects. CHD cases were more likely have a history of hypertension, diabetes mellitus (DM), and a family history of CHD. Total cholesterol (TC) levels were significantly lower in the cases than in the controls, possibly due to the use of cholesterol-lowering medications in this population.

**Table 1:** General characteristics of the study population.

Variables	Cases (*n* = 1146)	Controls (*n* = 1146)	*p* Value
Sex, m/f (%)	901/245 (78.6/21.4)	891/255 (77.7/22.3)	0.613
Age, years	60.5 ± 11.3	60.0 ± 10.3	0.23
Blood pressure, mmHg			
Systolic	133.6 ± 29.0	136.0 ± 25.3	0.034
Diastolic	82.0 ± 11.3	82.9 ± 15.2	0.11
Body mass index, kg/m^2^	23.7 ± 3.1	24.4 ± 3.3	<0.01
Fasting glucose, mmol/L	5.6 ± 2.2	6.2 ± 3.0	<0.01
Total cholesterol, mmol/L	4.35 ± 1.10	4.68 ± 0.92	<0.01
Triglyceride, mmol/L	1.61 ± 1.30	1.67 ± 1.38	0.283
Smoking, no/yes (%)	686/460 (59.9/40.1)	774/369 (67.7/32.3)	<0.01
Alcohol drinking, no/yes (%)	776/365 (68.0/32.0)	822/317 (72.2/27.8)	<0.01
Past history			
Hypertension, no/yes	784/361 (68.5/31.5)	351/790 (30.8/69.2)	<0.01
Diabetics, no/yes	1080/65 (94.3/5.7)	824/314 (72.4/27.6)	<0.01
Family history of CHD, no/yes (%)	1133/10 (99.1/0.9)	947/158 (85.7/14.3)	<0.01

Variables are presented as the mean ± SD or percentage. *p*-Values were calculated; using independent-sample *t*-tests or Chi-square tests.

### 2.2. Associations between Interleukin 33 (IL-33)/Interleukin 1 Receptor-Like 1 (ST2)/IL-1R Accessory Protein (IL-1RAcP) Variants and Coronary Heart Disease (CHD) Risk

All the SNPs conformed to Hardy–Weinberg equilibrium (HWE) (*p* > 0.001) except rs1157505, which significantly deviated from HWE in both control subjects and CHD cases (*p* < 0.001). The minor allele frequency (MAF) for -27307T/A and -27614C/A were 0, and these alleles have not previously been found in the Chinese Han population according to the HapMap database. Thus, 25 SNPs were selected for further analysis. To analyze the associations between these SNPs and CHD risk, we compared differences in the genotype distribution of each polymorphism between case and control subjects and analyzed the genotypes as dominant/recessive models. As shown in Table 2, homozygous rs4624606 variants (AA) had a significantly increased risk of CHD (OR = 1.85; 95% CI = 1.01–3.36; *p* = 0.045) compared to subjects with homozygous wild-type alleles (TT) after adjustment for conventional CHD risk factors, such as age, sex, smoking, drinking, BMI, triglyceride (TG), hypertension, DM, and family history of CHD. The SNP rs4624606 was also associated with a significantly increased risk of CHD in a recessive model (AA *vs.* (TT *+* TA): OR = 1.85; 95% CI = 1.02–3.35; *p* = 0.043, Table 2). This study had greater than 90% power to detect the associations between rs4624606 and CHD risk > 1.85 at a 0.05 significance level under both additive and recessive models. However, *p*-values for rs4624606 failed to reach significance after Bonferroni correction (*p* > 0.002). There was no significant association between the remaining 24 SNPs and CHD risk in the Chinese population (*p* > 0.05).

**Table 2:** Genotype frequencies of 25 SNPs and their association with coronary heart disease (CHD) risk in the Chinese population.

SNPs	Case ^a^	Control ^a^	Heterozygote ^b^		Variant Homozygote ^b^		Dominant Model ^c^		Recessive Model ^d^	
			OR (95% CI) ^e^	*p* ^e^	OR (95% CI) ^e^	*p* ^e^	OR (95% CI) ^e^	*p* ^e^	OR (95% CI) ^e^	*p* ^e^
*IL33*										
rs1929992	353/538/217	369/527/210	1.04 (0.82–1.32)	0.733	1.11 (0.83–1.49)	0.485	1.06 (0.85–1.32)	0.594	1.08 (0.84–1.41)	0.542
rs10975520	327/554/246	335/539/234	1.00 (0.79–1.27)	0.987	1.08 (0.81–1.44)	0.618	1.02 (0.82–1.28)	0.83	1.07 (0.84–1.38)	0.570
rs11792633	361/552/222	363/530/211	1.01 (0.80–1.28)	0.912	1.09 (0.81–1.45)	0.581	1.03 (0.83–1.29)	0.762	1.08 (0.83–1.39)	0.571
rs1624159	1010/94/3	1020/91/6	0.88 (0.61–1.28)	0.513	2.83 (0.58–13.9)	0.199	0.93 (0.65–1.35)	0.709	2.86 (0.58–14.04)	0.195
*ST2*										
rs3755278	974/86/3	1045/89/4	1.02 (0.70–1.49)	0.928	3.44 (0.53–22.19)	0.193	1.06 (0.73–1.54)	0.743	3.44 (0.53–22.16)	0.194
rs3821204	457/512/157	452/522/134	0.94 (0.76–1.18)	0.612	1.26 (0.90–1.75)	0.178	1.00 (0.81–1.24)	0.972	1.30 (0.95–1.77)	0.102
rs13431828	925/194/15	932/166/10	1.16 (0.88–1.54)	0.284	0.91 (0.35–2.38)	0.843	1.15 (0.87–1.50)	0.326	0.89 (0.34–2.32)	0.805
rs10206753	839/272/26	832/252/24	1.07 (0.84–1.37)	0.562	0.97 (0.49–1.93)	0.939	1.06 (0.84–1.34)	0.598	0.96 (0.48–1.89)	0.898
rs1041973	845/248/41	844/234/30	1.00 (0.78–1.29)	0.977	1.41 (0.79–2.49)	0.243	1.05 (0.83–1.33)	0.681	1.40 (0.80–2.48)	0.242
rs12999364	409/536/185	407/527/174	0.99 (0.79–1.24)	0.908	1.13 (0.83–1.55)	0.436	1.02 (0.82–1.26)	0.854	1.14 (0.86–1.51)	0.363
rs6543116	337/571/216	326/537/245	1.02 (0.81–1.30)	0.845	0.82 (0.61–1.10)	0.189	0.96 (0.77–1.21)	0.735	0.81 (0.62–1.04)	0.101
rs951774	660/413/58	630/408/70	0.91 (0.73–1.13)	0.377	0.91 (0.57–1.45)	0.683	0.91 (0.74–1.12)	0.358	0.94 (0.60–1.49)	0.8
rs10515922	843/263/26	833/247/28	1.00 (0.78–1.27)	0.976	1.13 (0.55–2.29)	0.745	1.01 (0.79–1.28)	0.955	1.13 (0.55–2.29)	0.742
rs13006559	1063/77/2	1046/59/3	1.24 (0.80–1.94)	0.34	1.37 (0.12–15.25)	0.798	1.25 (0.80–1.93)	0.326	1.36 (0.12–15.10)	0.804
*IL–1RAcP*										
rs1015704	725/359/46	686/381/41	0.89 (0.71–1.11)	0.295	1.02 (0.59–1.78)	0.935	0.90 (0.73–1.12)	0.34	1.07 (0.62–1.84)	0.819
rs9817203	515/493/122	506/489/113	1.05 (0.84–1.30)	0.685	1.24 (0.87–1.77)	0.228	1.08 (0.88–1.33)	0.461	1.22 (0.87–1.70)	0.255
rs1559018	300/549/279	276/569/262	0.97 (0.76–1.25)	0.825	1.14 (0.85–1.53)	0.378	1.02 (0.81–1.30)	0.846	1.16 (0.91–1.48)	0.223
rs3773986	861/245/19	844/250/14	1.01 (0.79–1.29)	0.936	1.23 (0.52–2.91)	0.644	1.02 (0.80–1.30)	0.86	1.22 (0.52–2.90)	0.647
rs6765375	736/350/45	699/375/34	0.89 (0.71–1.11)	0.301	1.18 (0.66–2.11)	0.576	0.91 (0.74–1.13)	0.408	1.23 (0.69–2.18)	0.486
rs4624606	734/338/46	731/351/26	0.99 (0.79–1.25)	0.962	1.85 (1.01–3.36)	0.045	1.05 (0.85–1.31)	0.636	**1.85 (1.02–3.35)**	**0.043**
rs16865597	877/204/12	854/223/17	1.02 (0.78–1.32)	0.892	1.19 (0.45–3.12)	0.728	1.03 (0.79–1.33)	0.837	1.18 (0.45–3.11)	0.733
rs4687150	559/434/105	543/433/110	0.92 (0.74–1.15)	0.459	0.90 (0.62–1.30)	0.58	0.92 (0.74–1.13)	0.412	0.94 (0.66–1.33)	0.712
rs3773958	380/548/200	368/534/206	0.97 (0.77–1.22)	0.779	0.95 (0.70–1.28)	0.729	0.96 (0.77–1.20)	0.728	0.97 (0.74–1.26)	0.806
rs3773981	695/330/57	726/336/42	0.94 (0.75–1.18)	0.59	1.09 (0.65–1.80)	0.751	0.96 (0.77–1.19)	0.692	1.11 (0.67–1.83)	0.689
rs6444435	638/340/65	691/363/51	0.97 (0.77–1.22)	0.797	1.39 (0.87–2.23)	0.168	1.02 (0.82–1.27)	0.843	1.41 (0.88–2.24)	0.150

^a^ Wild-type homozygote/heterozygote/variant homozygote; ^b^ The reference group comprised subjects homozygous for the wild-type allele; ^c^ Dominant model (wild-type homozygote *vs.* heterozygote + variant homozygote); ^d^ Recessive model (wild-type homozygote + heterozygote *vs.* variant homozygote); ^e^ Data were calculated by unconditional logistic regression, adjusted for age, sex, smoking, drinking, BMI, TG, hypertension, DM, and family history of CHD. Numbers in bold mean the SNP rs4624606 was also associated with a significantly increased risk of CHD in a recessive model (AA *vs.* (TT *+* TA): OR = 1.85; 95% CI = 1.02–3.35; *p* = 0.043, Table 2).

### 2.3. Association Analyses for Stratified Traditional Risk Factors

To evaluate whether traditional risk factors influenced the effects of genetic variants on CHD risk, we conducted stratification analysis for rs4624606 by sex, smoking, drinking, BMI, hypertension, and diabetes and examined the interactions between this SNP and traditional risk factors for CHD risk. As shown in Table 3, rs4624606 was associated with higher risk in hypertensive subjects (AA *vs.* TT: OR = 2.77, 95% CI = 1.01–7.57; AA *vs.* (TT *+* TA): OR = 2.91, 95% CI = 1.07–7.93). However, no significant interactions were observed between rs4624606 and sex, smoking, drinking, BMI, hypertension, or diabetes (all *p* > 0.05).

**Table 3:** Stratification analysis for the association between rs4624606 and the risk of CHD.

rs4624606 OR (95% CI) ^a^							
Variables	TT	TA	AA	*p* ^b^	TT + TA	AA	*p* ^b^
Sex							
Male	1.00	0.95 (0.74–1.22)	1.70 (0.89–3.23)	0.515	1.00	1.72 (0.91–3.27)	0.826
Female	1.00	1.24 (0.72–2.12)	1.37 (0.22–8.69)		1.00	1.26 (0.20–7.93)	
Smoke status							
Smokers	1.00	0.98 (0.67–1.45)	1.68 (0.58–4.88)	0.820	1.00	1.69 (0.59–4.87)	0.951
Nonsmokers	1.00	0.95 (0.72–1.26)	1.82 (0.87–3.80)		1.00	1.85 (0.89–3.84)	
Drink							
Drinkers	1.00	0.92 (0.60–1.39)	1.99 (0.61–6.43)	0.974	1.00	1.74 (0.86–3.53)	0.793
Nondrinkers	1.00	1.00 (0.76–1.30)	1.74 (0.85–3.55)		1.00	2.04 (0.64–6.56)	
BMI, kg/m^2^							
< 25	1.00	1.08 (0.82–1.43)	1.55 (0.70–3.47)	0.204	1.00	1.52 (0.68–3.38)	0.365
≥ 25	1.00	0.85 (0.58–1.24)	2.24 (0.88–5.75)		1.00	2.33 (0.92–5.91)	
Hypertension							
No	1.00	1.22 (0.89–1.68)	1.32 (0.57–3.05)	0.491	1.00	1.24 (0.54–2.84)	0.246
Yes	1.00	0.85 (0.62–1.17)	**2.77 (1.01–7.57) ^c^**		1.00	**2.91 (1.07–7.93) ^c^**	
Diabetes							
No	1.00	1.02 (0.80–1.29)	1.85 (0.99–3.44)	0.626	1.00	1.84 (0.99–3.40)	0.941
Yes	1.00	0.87 (0.42–1.79)	1.72 (0.17–17.27)		1.00	1.78 (0.18–17.72)	

^a^ ORs were obtained from an unconditional logistic regression model with adjustment for age, sex, smoking, drinking, BMI, TG, hypertension, DM, and family history of CHD. For each stratified analysis, adjustments were made for all other variables excluding the stratified variable; ^b^ *p*-Values of interactions were calculated by unconditional logistic regression; ^c^ *p* < 0.05.

## 3. Discussion

In this case-control study, we investigated the associations of 28 polymorphisms in IL-33/ST2 signaling pathway genes with the risk of CHD. Our results showed that subjects with the rs4624606 AA genotype in the *IL-1RAcP* gene had an increased risk of CHD. Especially among hypertensive subjects, the genotype AA was strongly associated with a higher risk of CHD. It is possible that hypertension may exacerbate the influence of genetic factors. No variants in *IL-33* or *ST2* were associated with CHD risk. Moreover, no interactions were observed between these SNPs and CHD traditional risk factors.

Numerous *in vitro* and *in vivo* model studies have found that activation of the IL-33/ST2 signaling pathway can significantly attenuate the severity of CHD [4]. IL-33 appears to provide anti-atherosclerotic benefits in CHD. Furthermore, the sST2 isoform acts as a decoy receptor to reduce IL-33 signaling through the ST2L receptor and is a significant predictor of mortality in patients with several cardiovascular disorders [6]. To further explore the role of the *IL-33* gene in the pathogenesis of CHD, we tested the association between *IL-33* polymorphisms and CHD; however, no significant associations were found, nor were there significant interactions between these SNPs and traditional CHD risk factors.

The *ST2* gene is located in chromosome 2q12 and contains 11 exons, a proximal promoter, and a distal promoter [24]. Many researchers have reported that rs6543116 (-26999G/A) in the distal promoter region of *ST2* is associated with increased risk for atopic dermatitis and asthma [32,34]. Recently, Tsapaki *et al.* reported that -27307T/A and -27614C/A polymorphisms in the distal promoter region of *ST2* influence susceptibility to severe CHD [20]. Based on these results, we hypothesized that SNPs in the distal promoter region of *ST2* might be associated with CHD risk. In this study, we replicated these SNPs (rs6543116, -27307T/A, -27614C/A) and examined another three SNPs (rs951774, rs10515922, and 13006559) in the distal promoter region, which were selected by extending 30 kb into the 5' end of *ST2*. We also analyzed the tagSNPs in the *ST2* gene to explore the genetic role of *ST2* in the pathogenesis of CHD. However, we failed to show an association between these SNPs and CHD. One possibility for this discrepancy may be due to ethnic and phenotypic differences and disparate environmental effects between countries.

IL-1RAcP is a member of the *IL-33/ST2* axis receptor complex and has been reported to play an important role in CHD [9]. Soluble IL-1RAcP interacts with the sST2-IL-33 complex to increase inhibition of IL-33 signaling. The *IL-1RAcP* gene is located on chromosome 3q28. Genetic variants in *IL-1RAcP* have been associated with prostate cancer, Kawasaki disease, and persistent hepatitis B virus [23,30,33]; however, the role of *IL-1RAcP* polymorphisms in CHD has never been investigated. In this study, we selected 11 tagSNPs and found that rs4624606 was associated with increased risk of CHD. Ramkumar *et al.* also reported that rs4624606 was associated with higher amniotic fluidIL-1β concentrations [26]. The SNP rs4624606 is located in the intron of *IL-1RAcP*. However, no functional studies have been performed to determine the characteristics of this SNP. Further research is required to understand the functions of variants associated with CHD.

Our study has several limitations. First, like other case-control studies, a possible selection bias (inclusion of surviving CHD patients) may exist. Second, not all controls had coronary angiography, introducing the possibility of false-negative cases. However, our controls had normal electrocardiography (ECG), no clinical symptoms, and no history of CHD before enrollment; additionally, false negatives are likely to be rare.

## 4. Experimental Section

### 4.1. Study Population

The sample population included 1146 patients and 1146 age- and sex-frequency-matched controls. CHD patients were recruited from three hospitals (Union Hospital, Tongji Hospital, and Wugang Hospital) in Wuhan, China [35,36]. CHD was diagnosed as angiographically demonstrated stenoses (≥50%) in a major or main branch of the coronary artery [37]. The control subjects, residing in the same communities as the patients, were determined to be free of CHD and peripheral atherosclerotic arterial disease by medical history, clinical examinations, and ECG. All subjects completed an Inter-Heart questionnaire and were interviewed about their demographic data, medical history, history of disease, family history of cardiovascular disease, and lifestyle habits (including smoking and alcohol consumption) by trained interviewers. The study was approved by the Ethics Committee of Tongji Medical College (project identification code: S073, 21 February 2011), and all participants provided written informed consent.

### 4.2. Selection of Polymorphisms

TagSNPs were selected based on the HapMap phase I & II database (http://www.hapmap.org, CHB and JPT as the reference set). According to the criteria of *r*^2^ ≥ 0.8 and minor allele frequency (MAF) ≥ 0.05, we extended 2000 bp into the 5' and 3' ends of *IL-33* and *IL-1RAcP*. To cover the distal promoter region of *ST2*, we extended 30 kb into the 5' and 2000 bp into the 3' end of *ST2*. Thus, we selected 18 tagSNPs in the three genes. We selected another 10 SNPs from previous reports in CHD (-27307T/A, -27614C/A) and other diseases, including Alzheimer’s disease, asthma, and atopic dermatitis [20,21,32,38]. In total, we selected and genotyped 28 SNPs (Table 4).

**Table 4:** SNP locations and allele frequencies.

SNP	Gene	Location	Genotype		MAF *		HWE *p* *	
rs1929992	*IL33*	Intron	A/G		0.489		0.655	
rs10975520	*IL33*	Intron	G/C		0.488		1	
rs11792633	*IL33*	Intron	T/C		0.433		–	
rs1157505	*IL33*	Intron	G/C		0.225		–	
rs1624159	*IL33*	Between genes	A/G		0.06		0.02	
rs3755278	*ST2*	intron	A/G		0.067		1	
rs3821204	*ST2*	3'-Flanking region	C/G		0.358		0.403	
rs13431828	*ST2*	5'-Flanking region	T/C		0.089		1	
rs10206753	*ST2*	Exon	C/T		0.133		1	
rs1041973	*ST2*	Exon	A/C		0.156		0.527	
rs12999364	*ST2*	Between genes	T/C		0.45		–	
rs6543116	*ST2*	Distal promoter	A/G		0.367		–	
rs951774	*ST2*	Distal promoter	A/C		0.233		0.251	
rs10515922	*ST2*	Distal promoter	C/T		0.151		1	
rs13006559	*ST2*	Distal promoter	T/C		0.07		1	
-27307T/A	*ST2*	Distal promoter	T/A		–		–	
-27614C/A	*ST2*	Distal promoter	C/A		–		–	
rs1015704	*IL-1RAcP*	intron	G/A		0.222		0.294	
rs9817203	*IL-1RAcP*	intron	T/C		0.395		0.439	
rs1559018	*IL-1RAcP*	intron	G/A		0.488		0.479	
rs3773986	*IL-1RAcP*	intron	T/C		0.067		1	
rs6765375	*IL-1RAcP*	intron	C/A		0.233		1	
rs4624606	*IL-1RAcP*	intron	A/T		0.213		0.294	
rs16865597	*IL-1RAcP*	intron	C/T		0.159		0.273	
rs4687150	*IL-1RAcP*	intron	T/C		0.341		0.403	
rs3773958	*IL-1RAcP*	intron	G/T		0.419		0.752	
rs3773981	*IL-1RAcP*	intron	C/A		0.182		0.584	
rs6444435	*IL-1RAcP*	intron	A/G		0.251		0.233	

* Data from the NCBI; – Data were not found in http://www.ncbi.nlm.nih.gov/snp/.

### 4.3. DNA Isolation and Genotyping

Fasting venous blood was collected from the peripheral vein, and genomic DNA was extracted with a Puregene kit (Gentra Systems Inc., Minneapolis, MN, USA). Twenty-five SNPs were genotyped using the Sequenom MassArray system (Sequenom Inc., San Diego, CA, USA). The remaining rs3755278, rs6444435, and rs1157505 SNPs were genotyped by TaqMan SNP allelic discrimination (Applied Biosystems, Foster City, CA, USA). TaqMan data collection and analysis were performed with SDS 2.2.1. Thus, 28 SNPs were successfully genotyped with a successful call rate of greater than 95%, with the exception of rs6444435, for which the call rate was 93.7%.

### 4.4. Biological Variable Determination

FBG, TC, and TG were assayed by standard laboratory procedures in the clinical laboratory department at Union Hospital.

### 4.5. Statistical Analysis

Continuous variables are expressed as means ± standard deviation (SD). The normal distribution of data was verified by the Kolmogorov–Smirnov normality test. Continuous data with a normal distribution were compared by Student’s *t-*test, and those with unequal variance or without a normal distribution were analyzed by Mann–Whitney rank-sum tests. Chi-square tests were used to compare categorical variables and the HWE of the polymorphisms. Unconditional logistic regression analysis was used to estimate the associations between SNPs and CHD risk after adjustment for age, sex, smoking, drinking, body mass index (BMI), TG, hypertension, DM, and family history of CHD by odds ratios (ORs) and 95% confidence intervals (CIs). The interactions of covariates with SNP genotypes were tested using the Wald test in unconditional logistic regression models. The significance of multiplicative interactions between SNPs and covariates was determined by the likelihood ratio test using the logistic regression model. Power calculations were performed using the QUANTO software program (Version 1.2.3) [39]. All other statistical analyses were carried out with the statistical analysis software package SPSS 12.0 (SPSS Inc., Chicago, IL, USA). Differences or associations with *p*-values of less than 0.05 were considered significant.

## 5. Conclusions

In conclusion, we found that the SNP rs4624606 within the *IL-1RAcP* locus was nominally associated with CHD susceptibility. Further studies in other independent populations with large sample sizes are required to validate these findings.
